# The impact of digital empowerment on the economic openness of the textile industry in Keqiao based on a sustainable environment

**DOI:** 10.1016/j.heliyon.2023.e23812

**Published:** 2023-12-17

**Authors:** Bofan He, Yao Chen, Nurlida Ismail, Gang Chen, Liheng Ni

**Affiliations:** a School of International Business, Zhejiang Yuexiu University, Shaoxing, 312000, Zhejiang, China; b School of Management & Marketing, Taylor's University, Subang Jaya, 47500, Selangor, Malaysia

**Keywords:** Keqiao, Digital empowerment, Sustainable environment, Economic development, Textile industry

## Abstract

Based on the framework theory of industrial digitization, digital industrialization, digital governance and digital value-added in a sustainable environment, this paper systematically studies the relevant elements of governance and value distribution in the sustainable environment of global trade, and its impact on the development path of human beings. This paper explores the way to embed digital technology into the global value chain to realize digital empowerment, measures the competitiveness index of Keqiao's textile industry in the global value chain, analyzes the technical and environmental challenges faced by China's textile industry in the digital age, and proposes the corresponding countermeasures to deal with the impact of global value chain participation and to improve the international competitiveness of China's textile industry.

## 1 Introduction

In recent years, in order to consolidate further the traditional advantageous position of developed countries in the field of manufacturing and enhance their market competitiveness, developed countries, led by Germany, have taken the lead in putting forward the Industry 4.0 plan, which largely restricts the advantages of production factors in related industries in China, making China face the pressure from both the transfer of industries from developing countries and the return of manufacturing industries from developed countries, which is closely related to our role in the global division of labor. In 2013, in response to this initiative of developed countries, China formally put forward the "manufacturing backflow plan", emphasizing the innovative use of artificial intelligence, big data, Internet of Things and other digital technologies in the traditional manufacturing industry to lay a solid foundation for the transformation and upgrading of traditional economic fields and innovative research and development. solid foundation.

Zhejiang Province is China's traditional strong manufacturing province, the output of its textile industry in 2020 were valued at 318.02 billion Yuan, among which, Keqiao District accounted for one third. As a typical industrial powerhouse, Keqiao District in Shaoxing, Zhejiang Province has experienced quite a few bottleneck problems such as a low level of technology in its textile industry, the lack of scientific management, the loose environmental pollution control of the production process, the incomplete industrial chain.

In terms of theoretical significance, based on the current development situation and problems of the textile industry in Keqiao District, Shaoxing, Zhejiang, this study analyzes and researches the development situation of the textile economy in the area from two perspectives: rationalization of economic development and heightened economic development, which helps to better apply digital technology to the transformation and upgrading of the textile industry in the area and provides theoretical reference for its industrial optimization and adjustment.

In terms of practical significance, it helps to integrate comprehensively and deeply the traditional textile industry and the new generation of digital technology, enhance the position of China's textile industry in the global value chain system, promote the transformation and upgrading of the textile industry and environmental protection compliance, provide implementable suggestions for digital empowerment in the textile industry.

The goal of this study focuses on the impact of digital empowerment on the economic development of the textile industry in Keqiao District through evaluating the integration level of the traditional textile industry with digital technology, measuring the economic development level of the region, and learning clearly the factors affecting the upgrading and transformation of the textile industry and digital empowerment. This study is based on the current development status and existing problems of the textile industry in Keqiao District, Zhejiang. It analyzes and studies the economic development status of the textile industry in this area, with the objectives of making both quantitative and qualitative analysis of the impact of digital empowerment on the textile industry economy in Keqiao.

## 2 Related theoretical foundations and literature review

### 2.1 Related concepts

#### 2.1.1 Digital empowerment

Don Tapscott, an American IT expert, defines digital technology as a basic general-purpose technology that integrates various communication infrastructures to achieve efficient and convenient transmission of information and data among different subjects. Other scholars identify physical information systems as the core of digital technology, which has undergone three evolutionary stages: informatization, digitalization and digital transformation. In the manufacturing industry, information technology is mainly manifested in the use of industrial Internet, big data and other technologies.

Specifically, the so-called big data technology refers to the technology related to the in-depth mining, comprehensive processing and effective analysis of massive data contained in distributed network architecture, database and other systems, and the timely uploading of the results obtained from the analysis to the terminal devices. This technology helps to precisely analyze the diversified needs of audience groups, achieve the effect of "data value-added", and realize the rapid processing and optimization of large quantities of data. Take Meituan as an example, it can accurately determine consumers' consumption preferences based on the price, purchase frequency and category of products booked by different consumers on the platform and use the results as an important basis for product recommendations to better meet the diversified needs of consumers.

To achieve digital empowerment, the essence is to effectively use various new digital technologies in the process of promoting traditional industries, that is, the digital development of traditional industries. In this process, the combination of traditional industries and digital technology should be analyzed in depth, and the development path of innovation and integration should be steadfastly pursued. Targeted transformation of enterprises of different types, scales and nature should be carried out, so that their production structure adjustment and product transformation and upgrading can highlight a higher level of digitalization and intelligence and be better aligned with market demand and industry dynamics. In this way, the relevant industries in the international market value chain of intelligent manufacturing can be ensured to linked to certain advantages, while for the connection of different links, including people, equipment, processes, services, etc. they also have certain intelligent capabilities, and thus promote the overall digital development process of traditional industries.

#### 2.1.2 Value chain theory

In the general industrialized production model, the "smile curve" theory can be used to explain the industrial division of labor. Specifically, countries at different levels of development are engaged in different divisions of labor in the international industrial chain, which are determined by their different positions on the curve. At the micro level, if any enterprise wants to obtain excessive profits in the economic field, it cannot be limited to low-value links, including manufacturing and other links with certain repetitive and mechanical characteristics, but should explore the high value-added links at both ends of the curve, and expand and extend in a targeted manner to enhance the added value.

**Fig. 1:**
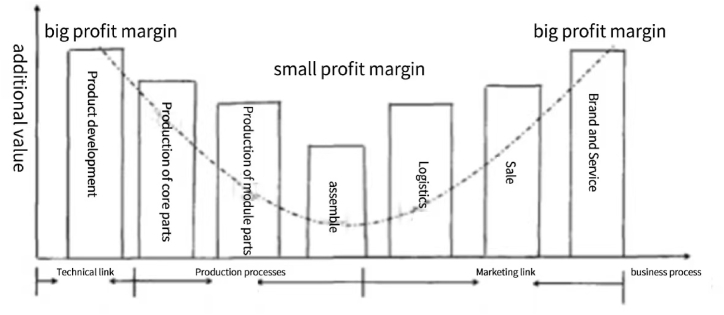
Schematic diagram of smile curve.

As indicated by the Fig. 1 above, the "smile curve" is represented by a U-shaped curve with a low center and high ends, with the horizontal direction representing the different production processes of economic agents from left to right, and the vertical direction representing the added value of different production processes. It divides an industrial chain into four processes: R&D, production, marketing and business, covering several interval modules. Most of the links represented by parts production and assembly belong to the lowest end of the curve, which means that they do not have high added value and high profits. Therefore, at this stage, if economic agents in this business process want to expand their profit space, they must move forward to the two ends of the technical links and marketing services. For different economic agents, the role they play in the industrial division of labor largely determines their profitability. From the level of the international industrial chain, economic agents in developed countries tend to occupy a high-end position in the industrial chain, which has a large profit margin and sustainability, and almost no marginal costs due to production in the later stages. Other economic agents, on the other hand, do not have significant advantages in terms of channels or technology, and have been at the lower end for a long time, and can only obtain limited profits through single, mechanical labor. In addition, these links for economic agents are not high, the substitutability between different subjects is strong, and thus the profit margin of economic agents in late-developing countries is further squeezed.

Along with the rapid development of a new generation of digital technology and its wide application in various fields, it has greatly changed the shape of the traditional smile curve, and the original steeper trend has gradually become flat. The fundamental reason for this is that digital technology has enabled traditional industries to adjust the value production and distribution mechanism of the original industrial value chain. In addition, with the empowerment of digital technology, different economic agents can widely participate in various links and business processes of economic activities, thus the problem of poor communication and less communication between different links can be effectively relieved, and the information asymmetry is greatly reduced, which makes the industrial value chain as a whole highlight the trend of flattening.

### 2.2 Literature review

#### 2.2.1 Digital empowerment related research

At the end of the last century, the rapid development of digital technologies such as the Internet and their widespread use in business gave rise to the e-commerce boom. Against this background, theoretical research on digital empowerment began. Mesenburg (1996) regrouped and categorized the impact areas of digital economy, and believed that software services, trading platforms, hardware could support the relevant electronic transaction infrastructure represented by various computer commerce activities such as email and video conferencing, and e-commerce itself, which together constitute the system of digital economy [1]. Bukht and Heeks (2017), on the other hand, argued that the digital economy can be divided into 3 levels based on the digitalization or service production need of a particular economic subject [2]. In particularly, the digital sector or subject sector covers industries such as information services and software production, while the digital economy also includes new business development models that have emerged because of the widespread application of relevant modern technologies in traditional fields, such as the digital service platform economy. The digital economy can be summarized as all economic activities based on digital technology, and this definition breaks the shackles of the traditional definition for the scope of the digital economy, indicating that digital technology has a broad pattern of future development and affects more fields.

#### 2.2.2 Research on the development trend of China's textile industry

In calculating the development of China's textile industry, Zhang & Wang (2019) used the index Herfindahl, which argued that China should prioritize the downstream part of the textile industry to shift out at the beginning of the 21st century, and gradually move upstream thereafter, and should fully shift out of the textile industry around 2010, as Japan did, and complete a sustained and effective transfer of the industry within 10 years [3]. Han & Cao (2018) believed that in the international value chain, the position of China's textile industry showed a steady rise. However, a geese formation industrial upgrading structure should also be implemented to break through the captive GVC development [4]. Huang and Nie (2016) point outed that Chinese textile and garment enterprises were at the low end of the global value chain, especially based on the increasingly intense and white-hot market competition pattern and increasingly stringent environmental protection requirements, they can only obtained certain advantages by lowering prices in the export process, which could easily fall into the "poverty growth "- the wrong path of development, relying only on lower environmental standards to maintain the scale of production to obtain meager profits, which was not a long-term solution [5].

#### 2.2.3 Research on the impact of digital empowerment on industrial economic development

Ulrich (2021) pointed out that there are significant fluctuations in the diffusion of technology in different fields, and the spillover characteristics of technology diffusion make it profoundly affect the economic transformation and upgrading in each field [6]. Kling & Lamb (1999) analyzed the return on investment in the information industry in 36 countries between 1985 and 1993 and argued that in the process of economic construction and development in developed countries, the construction of digital infrastructure and related technological innovation have a profound impact [7]. Gaputo & Marzi (2016) argued that the internet of things plays an important role in reshaping the industrial value chain and adjusting the industrial landscape in the process of economic development [8]. Huang & Yu (2019) pointed out that based on the information technology's sharing and spillover characteristics of information technology, information technology has a prominent role in the process of industrial restructuring and technological innovation development of enterprises [9].

Overall, the research of domestic scholars focused on the innovative concepts and methods brought by information technology and software service industry in the process of economic transformation and development. It believed that in the process of economic upgrading and development, information and software industries had given rise to many innovative digital technologies, which had greatly contributed to the adjustment of traditional production models and industrial patterns. Foreign scholars, on the other hand, paid more attention to the impact of economic development by the ICT industry. Ma & Meng & Yan pointed out that the effective use of digital technology in different fields had greatly contributed to the redistribution of traditional production factors, which helped to adjust the traditional production mode, optimized the distribution model, and enhanced the added value of different links [10]. Li & Sun (2020) selected 27 sub-sectors and systematically analyzed the development of China's manufacturing industry from 1990 to 2018 and analyzed that the main driver of its total factor growth rate lay in the effective application of digital technology. Through deep integration with the real economy, it has greatly reconstructed and optimized the production factor system and injected intrinsic momentum into the transformation and development of the economy [11]. Chi & Cai (2017) evaluated the current development of China's digital industry, which he believed was the necessary way to achieve the optimized and innovative development of traditional industries. Therefore, in China's manufacturing sector, it was necessary to adhere to the deep integration of traditional manufacturing and digital industries [12]. Ma (2021) showed that the new engine of manufacturing development lies in the integration and development with digital industry, which could empower traditional manufacturing industry and then provided support for the improvement of its output scale and the expansion of its efficiency [13]. Wang (2021) pointed out that the focus of China's industrial transformation and development at this stage was to promote the benign and sustainable development of the digital economy, further realize the all-round coverage of digital infrastructure, rationally allocate and optimize the use of resources, and solidify the digital technology foundation for the development of various industries [14].

### 2.3 Methodology

#### 2.3.1 Literature analysis method

In the course of this paper, we collected and collated relevant research literature on the impact of digital empowerment protocols on the economic development of textile industry in Keqiao District, Shaoxing, Zhejiang at home and abroad, and after careful consideration and analysis, we gradually strengthened our understanding of digital empowerment protocols and Zhejiang Keqiao District, and formed a framework of the impact of digital empowerment on the economic development of textile industry in Keqiao District, Shaoxing, Zhejiang, which theoretically provides the theoretical basis for this study.

#### 2.3.2 Statistical analysis method

The economic development data, trade data and investment amount of the textile industry in Zhejiang Keqiao District were analyzed through various literature, classifying different economic indicators and trade development indicators. At the same time, the current situation of the economic development of textile industry in Keqiao District, Shaoxing, Zhejiang, as well as its advantages and problems were analyzed in depth to provide data support for the research of this paper.

#### 2.3.3 Case study method

A case study method was used to carry out the description of the economic development of the textile industry in Keqiao District, Shaoxing, Zhejiang. Take Keqiao's textile industry as the specific research object, conduct in-depth investigation and analysis on its successful industrial experience and failure lessons with regards to the impact of the digital empowerment, and summarize the theories and methods that can be used for reference by the textile industry and other industries.

## 3 Keqiao District digital empowerment and industrial economy development status

### 3.1 Keqiao District digital empowerment status

#### 3.1.1 Value chain enhancement aspect

At present, China's comprehensive, in-depth supply-side reform and the increasingly stringent requirements for environmental protection related to industrial development, which puts forward higher requirements for the quality of product production and production process and the environmental standards of the products themselves. The implementation of the traditional textile industry concentrated in the low value-added surplus products and high environmental pressure production processes were generally eliminated, requiring the production of products with higher added value and environmental friendliness. This required the local government to create a good industrial development environment, guide all types of enterprises in the product development and production process into modern environmental technology, in-depth exploration of the cultural connotation of the product. In recent years, the government of Keqiao district has implemented these specific requirements as an opportunity to deepen the integration of various industrial development ideas, it planned and built a Jin Keqiao Science and Technology City, Science and Technology Park and other modern industrial parks, it also introduced a large number of innovative technologies to enhance the textile industry in all aspects of automation, standardization and modernization and environment-friendly level of the textile industry. In the process of product production, it introduced and applied various advanced equipment in all aspects, so that the manufacturing level of enterprises can be steadily improved, and the product upgrading can match the market demand. At the same time, Keqiao District tried to create high-quality fashion activities and strived to highlight the scale and benefits of various types of activities, so that the main bodies of the economy shifted gradually from pure product sales to the outward provision of high value-added creativity. In 2017, the scale of revenue obtained by related enterprises due to the textile creativity reached 1.2 billion Yuan, an annual increase of 20.55 %, while a sales scale of nearly 25 billion Yuan was achieved. In the process, the local government had introduced a series of policies to encourage the introduction of technology and R&D in all types of enterprises. The average annual increase in investment in technological reforms of relevant enterprises was as high as 10 %, and the proportion of textile equipment that was close to or had reached the internationally advanced level was over 90 %. At the same time, all types of enterprises had built up RRP management systems with wide coverage and perfection, laying a solid foundation for their internal information management.

#### 3.1.2 Marketing chain expansion aspect

In recent years, with regards to the marketing chain expansion, Keqiao District government has created vigorously a good market environment, all-round optimization of hardware supporting facilities, improved the level of hardware services. It has built or reconstructed the South Market, curtain and yarn market, storage and logistics center and other supporting markets and related facilities and plans to build a new China Textile City International Fine Market, which largely demonstrated its determination to develop industrial integration. At the same time, with the approval of the State Council, the textile expo held in the area has been promoted to a national exhibition. At this stage, China Textile City covers a floor area of 720,000 square meters and has registered nearly 10,000 companies with sales networks in nearly 200 countries or regions around the world, with an annual trading scale exceeding 140 billion Yuan.

In addition, the government of Keqiao District has also grasped firmly the network trend of industry development, promoted vigorously the development of network trading as well as cross-border e-commerce, created a good virtual market environment, and built up a perfect marketing network system for textile products. At this stage, the district has built and put into use five e-commerce parks, attracting more than 700 cross-border e-commerce enterprises, more than 7000 Alibaba "integrity pass" users, registered more than 80,000 business entities, opened more than 600,000 online stores. The local government is vigorously expanding the marketing chain on the network platform, so that its marketing coverage is increasingly expanding.

### 3.2 Current status of economic development in Keqiao District

The textile industry in Keqiao District is the mainstay of local economic development, accounting for 2/3 of the total scale of the region's industrial economy. At this stage, Keqiao District's annual chemical fiber production was more than 2.6 million tons, there are more than 8000 textile enterprises. Its production of chemical fiber and printing and dyeing cloth accounts for 10 % and 30 % of national outputs, respectively [15]. It can be said that the biggest traditional advantage of Keqiao District is the textile industry. At this stage, it has built up a relatively complete and orderly industrial chain as well as production and marketing system, which lays a solid foundation for the steady improvement of its industrial value.

In 2020, the scale of the large industrial output value of textile in the region is 109.79 billion Yuan, accounting for 52.9 % of the total output value of its industrial output. At the same time, under the premise of controlling the emission of environmental pollutants, the scale of output value of its printing and dyeing industry increased by 5.1 % annually [15]. Thus, the textile industry in the region has a steady and good development trend, which also laid a solid foundation for the improvement of the level of industrialization and the rapid development of the industrial economy in the region.

Textile industry is both the leading industry and pillar industry in Keqiao District. In recent years, Keqiao District around the construction of world-class modern textile industry cluster goals, adhered to the extension of the industrial chain, strengthened the innovation chain, enhanced the value chain, optimized the ecological chain. The integration of the four chains promoted vigorously the development of the textile industry from a traditional low-end survival to the high-quality development.

In terms of strengthening the innovation chain, Keqiao District completed the construction of the talent business park (Jin Keqiao Science and Technology City), the successful establishment of the provincial printing and dyeing manufacturing innovation center, gathered 8 printing and dyeing high-end engineers, including printing and dyeing engineers Collaborative Innovation Center and Modern Textile Jianhu Laboratory. Innovation Platform and "ten private talents entrepreneurship parks". At present, the region has 135 high-tech textile enterprises, 29 provincial level high-tech enterprise research institutes and 5 provincial level enterprise research institutes, which have comprehensively improved the level of basic research and industrial chain of the textile industry [16].

**Fig. 2:**
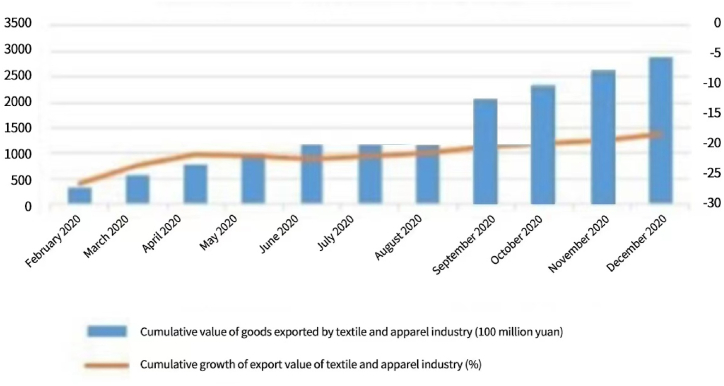
Keqiao district in 2020, textile and Apparel, Jewelry export delivery.

As indicated by the Fig. 2 above, in 2020, the Light Textile City achieved a transaction volume of 216.325 billion Yuan, an annual increase of 8.15 %; at the same time, the transaction scale of its online market trading platform built in that year was 60.75 billion Yuan, an annual increase of 15.28 %, Keqiao District registered newly 4676 companies, including 1488 newly registered trading companies, demonstrating fully the development potential of the industry and the confidence of these companies in the development of the industry [16].

(Data Source: BOSI Data Research Center)

## 4 The impact of digital empowerment on the economic development of textile industry in Keqiao District, Shaoxing, Zhejiang

### 4.1 Variable selection and data sources

The degree of economic openness in Keqiao District, Shaoxing, Zhejiang Province was analyzed as a dependent variable.

The five selected independent variables were the level of foreign economic development, industrial concentration, level of human capital, level of marketization, and digital empowerment. The degree of dependence and integration of a country or region in the international economy, i.e., the degree of economic openness is related to the level of foreign economic development, industrial concentration, the level of human capital, the level of marketization, and digital empowerment. Numerous previous studies have shown that these five factors (independent variables) have a decisive impact on the degree of economic openness in a region [3]. The specific meanings of these 5 independent variables are explained in detail below.

#### 4.1.1 Level of economic development

The level of economic development refers to the total wealth gained from economic development of a country or region in a certain period. However, in view of the difference in economic scale in Keqiao District, Shaoxing, Zhejiang, this paper proposes to use GDP per capita to reduce the impact of the difference in development scale.

#### 4.1.2 Industrial concentration

Industrial concentration is an indicator of the degree of aggregation of a certain industry in a certain region, and Shen Hongliang et al. (2020) argued that specialized production by means of industrial aggregation can, on the one hand, realized the rational allocation and optimal use of different factor resources, thus expanding the scale effect. On the other hand, it could also strengthen internal competition and technological spillover, and injected new vitality into the upgrading and development of industries. Thus, in the process of economic transformation and development at this stage, industrial concentration has a very profound impact. However, it should be noted that excessive aggregation can also cause waste of relevant factor resources. Thus, from an overall perspective, economic development is influenced by industrial concentration with uncertainty. The level of industrial concentration is measured by locational entropy in the process of this study.

#### 4.1.3 Human capital level

The level of human capital reflects the quality level of labor force in a region, and the index of average years of education is used in this study to indicate the level of human capital in the region. The formula is: human capital level = (number of elementary school graduates 6* + number of middle school graduates 9* + number of high school graduates 12* + number of higher education 16*)/total number of people.

#### 4.1.4 Marketability level

The level of marketization refers to the vitality of the development of market economy in a certain country and region under the conditions of a full market economy. A high level of marketization often means that the government no longer relies excessively on political means to control and regulate the economy, but rather regulates itself according to the spontaneous rules of the market, allowing for privatization of property rights or other changes according to certain rules. Under market-based regulation and control, the efficiency of resource allocation will be effectively improved, which can better promote economic growth. In this study, given that the statistical dimensions of Hong Kong and Macau are different from those of China, we use the indicator of the share of non-state enterprises and collective enterprises in total employment to measure the level of marketization, provided that data are available.

#### 4.1.5 Digital empowerment

During this study, reference was made to the white paper on China's comprehensive digital development released by the China Academy of Information and Communication and the research results of IDC on the information society index, and on the basis of comprehensive assessment and consideration of the collectability, continuity and systematization of relevant data, relevant data were collected in depth and comprehensively organized to build up an index system for evaluating the level of digital technology development in Zhejiang Keqiao District, identifying the digital infrastructure. The three primary indicators are digital infrastructure, digital technology application industry, and intelligent manufacturing industry, and 11 secondary indicators are selected under them, so as to measure the development level of information technology in the area in all aspects. (see Table 1)

One of the main indicators used to evaluate the construction of digital infrastructure is the internet penetration rate, the length of fiber optic cable lines, and the number of increased access ports. Generally, the larger the value of these indicators, the better the digital infrastructure coverage of the region is proved. For the evaluation of digital technology application, the output value indicators of electronic information manufacturing and information technology service industries were selected, thus this indicator can reflect the development benefits of digital technology in the region in a more objective and comprehensive way. Finally, the five major sub-industries in the textile industry are classified as smart manufacturing industries according to the definition of the core industry classification of the digital economy (2021) to show the reality of the digital efficiency improvement in the textile industry.

### 4.2 Model design and related tests

As indicated by the Table 2 above, in order to test the effects of economic development level, industrial concentration, human capital, marketization level and digital empowerment on the economic openness of Keqiao District, Shaoxing, Zhejiang, the following regression analysis model was established.

$$\ln\;F_{it}=\beta_{0}+\beta_{1}\;\ln\;{ES}_{it}+\beta_{2}\;\ln\;{IC}_{it}+\beta_{3}\;\ln\;{IF}_{it}+\beta_{4}\;\ln\;{HC}_{it}+\beta_{5}\;\ln\;{GE}_{it}+\varepsilon_{it}$$

**Table 1:** Digital empowerment level indicator system.

Indicator System	Primary Indicators	Secondary Indicators
Digital technology development	Digital Infrastructure	X Internet penetration rate (%)
		X Length of fibre optic cable lines (km)
		X Number of Internet broadband access ports
	Digital technology application industry	Software and information technology services revenue (million Yuan)
		electronic information manufacturing industry (billion Yuan)
	Intelligent manufacturing industry	X telecommunications services industry (billion yuan)
		intelligent equipment manufacturing industry (billion Yuan)
		X general equipment manufacturing industry (billion Yuan)
		special equipment manufacturing industry (billion Yuan)
		transportation equipment manufacturing industry (billion Yuan)
		X electrical machinery and equipment manufacturing industry (billion Yuan)
		X instrumentation manufacturing industry (billion Yuan)

**Table 2:** Variable descriptions.

Study variables	Metrics	Theoretical Implications
Economic Development Level	GDP per capita	The scale, speed and level of economic development of a country or region
Industry Concentration	Zone entropy	Reflects the level of industrial agglomeration in a region
Marketability Level	Employment in non-state and collective economy enterprises/total employment	Reflects the level of development of regional economic marketization
Digital Empowerment	Composite score of indicators	Indicates the level of digital technology development in a region
Human capital level	(6*number of elementary school education + 9*number of junior high school education + 12*number of senior high school education + 16*number of higher education)/total employment	Reflecting the quality of a region's workforce

In the above regression analysis model, F denotes the economic openness of region i in year t, ES denotes the level of economic development in region i in year t, IC denotes the industrial concentration in region i in year t, IF denotes the level of marketability in region i in year t, GE denotes the digital empowerment in region i in year t, and HC denotes the level of human capital in region i in year t. In addition, β0 is a constant term, β1, β2, β3, β4, and β5 are parameters to be estimated and are random disturbance terms.

The panel data of each area in Keqiao District, Shaoxing, Zhejiang from 2002 to 2021 was collected, compiled during this study, and the data was obtained from the Online Zhejiang Statistical Yearbooks on the official website of Zhejiang Provincial Bureau of Statistics in April 2022.

The statistics software of STATA was used to make the all the following statistical analysis and regression analysis as indicated by the Table 3 below.

**Table 3:** Descriptive statistics results.

Variables	Average	Standard deviation	Maximum	Minimum
Economic Development Level ES	1.840542	0.950465	4.003885	−0.298150
Industry Concentration IC	−1.780008	1.568686	1.257688	−5.141540
Human Capital Level HC	1.473612	0.037556	1.565325	1.400088
Marketability Level IF	−0.448438	0.377182	−0.056934	−1.550596
Digital Empowerment GE	1.652386	0.525300	3.009847	1.333008

Before carrying out the regression analysis work, the correlation, unit root, and cointegration tests of the data of interest were first passed, and the characteristics of the data of interest were presented.

As indicated by the Table 4 above, it can be seen that the absolute value space of the correlation coefficients of economic development level, human capital level, marketization level, industry concentration and system quality in the empirical model constructed in this paper is 0∼0.8. The correlation degree is obviously low, thus it can be considered that there is no multicollinearity in this model.

**Table 4:** Correlation test results.

Variable Name	ES	IC	HC	IF	GE
ES	1.00	0.28	−0.30	0.35	0.22
IC		1.00	−0.22	0.10	0.12
HC			1.00	−0.20	−0.24
IF				1.00	0.01
GE					1.00

To verify the stability of the panel model data, the LLC test and the ADF test were used to validate all data sets, and the SIC criterion was chosen to determine the delay. The test results in Table 4 and Table 5 showed that not all original variable data sets pass the unit root test, but after first-order differencing, all data passed the ADF test, as well as the Johansen system cointegration test, thus effectively avoiding the possibility of spurious regression of serial data. As a whole, all models passed the correlation test, and thus there was cointegration relationship between the variables of the models used in the course of this study.

**Table 5:** Unit root and Co-integration test results.

	Original sequence		Original sequence	
	LLC (t Value)	ADF (* Value)	LLC (t Value)	ADF (* Value)
F	−3.28***	26.68	−10.23***	102.16***
ES	0.61	9.17	−4.37***	41.86***
IC	−2.20**	35.08**	−7.95***	90.10***
HC	1.80	4.49	−9.66***	79.91***
IF	−2.84***	25.09	−9.41***	78.15***
GE	0.69	11.16	−7.04***	49.62***
cointegration test	ADF Statistical quantities		−33.89***	

Note: ***, ** and * indicate passing the 1 %, 5 % and 10 % significance level tests, respectively.

### 4.3 Analysis of empirical results

After the above tests, this study regressed the fixed, mixed, and random effects of the measurement equation to determine the most suitable one for regression. On this basis, Haussman and f-tests were conducted, respectively. The f-test was conducted in selecting the fixed and mixed effect models, and the resulting test statistic was 3694471 with a p-value of 0. In selecting the fixed and random effect models, Haussman test was conducted, and the resulting test statistic was 43.892391 with a p-value of 0. The results were therefore significant, so the final fixed effect model was used as the measurement equation for fixed effect regression.

As indicated by the Table 6 above, the regression coefficient of "industrial concentration" was significantly positive, and the 1 % significance level test showed that every 1 % increase in economic development level will lead to an increase in economic openness of about 1.7 %. Every 1 % increase in industrial concentration will lead to an increase in economic openness of about 2.6 %. Every 1 % increase in human capital level will lead to an increase in economic openness of about 19 %. Every 1 % increase in marketization level will lead to an increase in economic openness of about 1.6 %. Every 1 % increase in digital empowerment will lead to an increase in economic openness of about 13 %. The regression coefficient of the constant term is −4.3758, which is a constant and has no effect on the degree of economic openness of non-independent variables. Therefore, in regression analysis, the constant does not have much significance, so no matter what value appears, it does not need to be considered.

**Table 6:** Regression results.

	Regression coefficient	T Value
Economic Development Level	0.1743***	3.01
Industry Concentration	0.2623***	14.01
Human Capital Level	1.9002***	3.82
Marketability Level	0.1586*	1.84
Digital Empowerment	1.2987***	19.10
Constant term	−4.3758***	−4.43
R2	0.91	
Adjustment R2	0.90	
F Value	81.29***	

The regression coefficient of "human capital level" was greater than 0 and passed the 1 % significance level test. The results of domestic and foreign scholars showed that the human capital structure with only tertiary education often failed to play a significant role in improving the efficiency of research and innovation. Human resources are especially important today, as economic development is changing from financial and creative industries to sunrise industries, human resources not only directly lead the transformation and upgrading of primary and secondary industries, but also are closely related to the formulation of development plans, clarification of development directions and expansion of development paths of tertiary industries.

The regression coefficient of "marketization level" was positive, and the significance test was 10 %, that is, the higher the marketization level of the city of Keqiao District, Shaoxing, Zhejiang, the higher the degree of economic openness of Keqiao District, Shaoxing. The improvement of the marketization level of a region profoundly affects the overall openness of the regional economy and the development efficiency, so it is necessary to comprehensively improve the allocation efficiency of its resources and overall production efficiency.

The regression coefficient of "digital empowerment" was positive and the significance test was 1 %, it means that the higher the digital empowerment of the city of Keqiao District, Shaoxing, Zhejiang, the higher the degree of openness of the economy of Keqiao District, Zhejiang. Every 1 % increase in the level of digital technology development could bring about a 13 % increase in economic openness, and the role of economic openness enhancement was greater than that of economic openness rationalization, and from the standardized coefficient, the level of development of the digital economy was more important than the rationalization and heightening of economic development. Therefore, through the vigorous development of digital industry, through digital technology to the traditional textile industry empowerment, the textile industry digitalization, the economic development and upgrading of the textile industry can be realized.

### 4.4 Suggestions

#### 4.4.1 Active adjustment of China's textile industry

In order to achieve the optimization and adjustment of the industry as a whole, firstly, in product design, we should innovate design elements, increase the protection of design copyright, and stimulate the enthusiasm and subjective initiative of relevant subjects in design innovation, so as to resolve effectively the tendency of homogenization in the field of low-grade products, make them have a high cultural added value, and create independent brands that highlight Chinese characteristics. Secondly, in the production of products, the relevant economic entities should be encouraged to implement active mechanization, industrial production requirements, and promote its development in the direction of automation, low energy consumption and environmental friendliness. Finally, in the product sales chain, the national level government should increase policy guidance and capital investment, promote the implementation of trust and investment funds and other projects to relieve effectively local financial pressure while expanding offline sales channels for products, creating a good economic environment for physical stores, and providing the necessary support for high-quality offline market operations. Online, it is necessary to regulate further the means of operation of e-commerce companies, deal promptly with price wars and other malicious competitions, and promote the upgrading of the terminal market of large garment enterprises to build their own brands.

#### 4.4.2 Optimize digital empowerment policy

Optimization of digital empowerment policy includes mainly the following four aspects. First, from the level of digital industrialization, compared with the development of the software industry, the internet industry, etc., China should promote the continued development of the telecommunications industry in all aspects, to achieve comprehensive coverage of telecommunications base stations, so as to help residents effectively control the cost of communications, the formation of a development pattern based on a large domestic cycle, showing the huge potential contained in the domestic market, to drive the transformation and upgrading of the textile industry. Second, from the level of industrial digitization, it should further highlight the advantages of interaction, learn actively from, and apply the experience of western digital applications, and encourage entities to realize digital transformation in internal management, marketing, design, and publicity. Finally, in terms of digital governance, the cooperation between government departments and private entities should be further strengthened to create a comprehensive digital service system, to create a favorable external environment for the prevention of social risks and the provision of social services. In addition, from the perspective of digital valorization, emphasis should be placed on the protection of intellectual property rights and the establishment of more data centers to provide data security for the upgrading of functions and differentiated services of different enterprises.

#### 4.4.3 Optimize international trade and international investment environment

In the process of promoting the transformation and development of the textile industry, we should pay close attention to the trend of international trade and create a good international investment environment. Firstly, we should consolidate the traditional advantageous position of China's textile industry, implement deeply the development strategy of "one belt and one road", deepen the targeted trade with different countries in Southeast Asia, so as to consolidate further its core position in the countries along the "One belt and One road". At the same time, we should carry out actively cooperation with Central and Eastern Europe and other countries, such as the implementation of the "17 + 1″ dialogue mechanism, so that China's textile industry can have a visible advantage in the international market to create a broader platform for its transformation and upgrading. Secondly, we should enhance the position of pertinent companies in the global value chain in all aspects through in-depth industrial upgrading. Especially in response to some unfriendly measures taken abroad in recent years, the government must provide support for the external development of enterprises from the macro level, to create a gathering advantage of China's textile industry enterprises in the global value chain to achieve the dual upgrading of industry and enterprise. Thirdly, we should guide international investment in a targeted manner to help the textile industry's industrial upgrading. Specifically, we should actively guide the layout and investment of foreign capitals and encourage their inflows to the high-tech industries in the eastern China, especially the digital industry, to promote the upgrading and development of China's high value-added industries. At the same time, certain foreign investment can also be introduced in the central and western parts of the country to take advantage of the inexpensive labor force there, to inject vitality into the development of the textile industry.

## 5 Conclusions

Two variables, the level of digital technology development and industry concentration, clearly influence the heightened level of economic development of the textile industry in Zhejiang Keqiao District. And from the viewpoint of importance, the level of education and the level of digital technology development affect profoundly the heightened level of economic development of the textile industry in the region. With the help of the principal component analysis, it can be found that the development of digital industry is the main factor affecting the level of digital technology development, especially the development of information technology service industry and software service industry among them. Therefore, the transformation and upgrading of the textile industry in Keqiao District, Shaoxing, Zhejiang Province must be based on digital industrialization, thus empowering the traditional textile industry to improve the added value of each link of the industrial chain and expand its profit space under the premise of minimizing pollutants and environmental protection in the production process with high standards, so that China's textile industry has a higher position in the global industrial chain and finally realizes its industrial upgrading. The conclusions are quite new in a comparison with previous literature.

Despite the quite positive conclusion above, there are limitations all the same in this study, such as the insufficient inclusion of more independent variables or different independent variables affecting also the dependent variable in the regression analysis. Furthermore, there remains current research dilemmas in terms of the applicability of the digital empowerment on the economy of the textile industry of Keqiao District in other regions where the textile industry is also a pillar industry with quite different levels of economic development, industry concentration, human capital, marketability. Therefore, the future research directions may include similar examinations of the impact of digital empowerment on the economy of the textile industry in other regions in both China and other countries with additional independent variables, or different independent variables which are more relevant to the dependent variable.

## Funding statement

No funding was used to support this study.

## Data availability statement

This paper used data from the Online Zhejiang Statistical Yearbooks (2002–2021) on the official website of Zhejiang Provincial Statistics Bureau at http://tjj.zj.gov.cn/col/col1525563/index.html, which is publicly available in the Chinese language, anyone who is interested in checking the data used in this paper can refer to the website above. The same data in English is also available at https://weibo.com/mygroups?gid=110007884021796, no accession number is needed.

## Ethics declarations

Review and/or approval by an ethics committee was not needed for this study because this paper includes no content related to ethical issues.

## Additional information

No additional information is available for this paper.

## CRediT authorship contribution statement

**Bofan He:** Validation, Supervision, Methodology, Conceptualization. **Yao Chen:** Writing – review & editing, Writing – original draft. **Nurlida Ismail:** Methodology. **Gang Chen:** Software, Formal analysis. **Liheng Ni:** Resources, Investigation, Data curation.

## Declaration of competing interest

The authors declare that they have no known competing financial interests or personal relationships that could have appeared to influence the work reported in this paper.
